# Re-annotation of the sequence > annotation: opportunities for the functional microbiologist

**DOI:** 10.1111/1751-7915.12242

**Published:** 2015-01-28

**Authors:** Francisco Barona-Gómez

**Affiliations:** Evolution of Metabolic Diversity Laboratory, Unidad de Genómica Avanzada (Langebio), Cinvestav-IPNKm 9.6 Libramiento Norte, Carretera Irapuato – León, Irapuato, Guanajuato, CP36821, México

Functional annotation of proteins has been central to the development of biology in the post-genomic era. In such a way, the wealth of information encoded by genome sequences has become accessible to the broader biological community. One may even argue that this has served the purpose of democratization of science, as almost every scientist in the world has access to both genetic public databases and the little computing power needed for doing similarity Blast searches. However, I will argue here that this framework is flawed as it sticks to the once very useful, but now limited and simplistic assumption, that ‘anything found to be true of *E. coli* must also be true of elephants’, a famous statement by Jacques Monod around half a century ago.

In the most common annotation process, we label biomolecules, coded for in any given genome now routinely sequenced even by small laboratories, with a functional attribute. Annotation relies on sequence similarity searches and in what is known about the molecular biological functions of similar biomolecules in diverse organisms. As the latter knowledge, for instance the glycolytic pathway, was first obtained in model organisms such as *Escherichia coli*, functional annotation is about detecting (remote!) homologues using sensitive bioinformatics algorithms, and subsequent propagation of functional ‘experimentally validated’ data from the well-known model organisms, to our distantly related subject of study.

But what are the limitations associated with the current and broadly accepted approach used for functional annotation? And more importantly, what may be future research opportunities for the field and for the new generation of ‘functional microbiologists’ armed with both computational and wet laboratory experimental tools? Providing some preliminary answers to these questions is what I will aim at in this piece.

At least two problems can be envisioned when carefully considering the current conceptual functional annotation workflow. First, how certain are we about the original function found in the closest model organism to our subject of study? Is this function actually accurate and complete? In other words, is it safe to state that enzymes and proteins stick to the co-linearity principle of one gene – one protein – one function? Paradoxically, probably not a single molecular biologist nowadays will stand up for this principle, but we all assume it is correct when it comes to functional annotation of our genomes! Second, how safe it is to assume that what is true for one organism is true for another organism with a different evolutionary history? How could we account for biodiversity, which is at the core of traditional and modern biological thinking, as it is to evolutionary processes? For the sake of simplifying the analysis of biological systems, how far should the universality argument be put forward?

The answers to these questions have to begin by criticizing the simplistic conceptual framework that has prevailed to date in functional annotation. Not even as a reasonable starting point, as colleagues have challenged me when expressing these concerns, can we continue to accept this framework. Simply, among other reasons, because it is wrong and we can do better: our current understanding of enzyme promiscuity (Khersonsky and Tawfik, 2010) and ‘moonlighting proteins’ (Piatigorsky, 2007) provide an ideal scenario to showcase what is wrong and how we can do better.

Proteins and enzymes are for the most part believed to be functionally highly specific. However, enzyme promiscuity, which can be defined as the ability of an enzyme to catalyse chemical conversions in addition to the one they have primarily evolved for – using the same active site – is pervasive. Moreover, the functional diversity of proteins is further expanded by their ability to perform more than one activity, for instance, a physical interaction within a regulatory network in addition to a chemical conversion. This observation has led to the appearance of the term moonlighting proteins, which aims to account for the functional ephemeral nature of proteins.

The field of evolutionary biology has been responsible for advancing these concepts. The redundancy of enzymatic and protein functions has been hypothesized to lead to robust yet ‘plastic’ metabolic and regulatory networks, important for exploring metabolic diversity and organismal evolution. At the protein level, moreover, these ‘secondary’ activities have been hypothesized to serve as raw material for the evolution of new functions. Although specialization seems the ultimate outcome of evolution, most current evolutionary biologists will embrace enzyme promiscuity and moonlighting proteins as evolutionary advantageous, and they will likely agree that these phenomena are part of a wider mechanism for appearance of functional novelty and microbial adaptation.

Communities outside the subdiscipline of enzyme and protein evolution, unfortunately, seem not to have grasped these concepts. Indeed, I will argue that none of us annotating genomes have done so, posing a fundamental threat to the development of our own research activities. From microbial biotechnology to environmental microbiology, in a daily basis, we heavily rely on analyses of large sequence datasets derived after one or many of the omics technologies. When doing so, trying to come up with testable functional hypotheses that can be inferred from the sequences being functionally annotated, one may ask how many experiments have actually failed because of neglecting enzyme promiscuity and moonlighting proteins.

And here is where the opportunities for the functional microbiologist may arise. Metabolically speaking, enzymes do not exist as independent and autonomous entities. Their biological *raison d'etre* will only be accomplished when they become part of a metabolic pathway or even an entire metabolic network. The contrary also stands true; pathways and networks cannot exist without all their key components properly accounted for, i.e. functionally annotated. The field of metabolic modelling from genetic data has witnessed substantial progress in the last three decades (Bordbar *et al*., 2014), and beyond the applications in metabolic engineering and systems biology, embracing these tools for molecular functional annotation does provide a much needed and very interesting opportunity.

Computationally speaking, to start with, the modern biochemist annotating genomes should be able to assess the enzyme functions of all predicted proteins encoded by a genome beyond sequence similarity searches. Protein structural predictions, together with active site architecture and ligand binding molecular docking predictions (Skolnick *et al*., 2013), may indicate potential substrate and cofactor specificities. Genomic context and phylogenetic occurrence, together with gene expression and text-mining data, may suggest functional associations and interactions between proteins (Franceschini *et al*., 2013). These are just some examples showing that the conceptual framework for such annotation approach is already available.

So what may arise in the future are annotation tools that will allow integrating different layers of information in a simplified fashion. The aim should be to have a glimpse of the metabolome of all microbial types as part of its functional annotation. For this purpose, genome sequences in the future will be submitted to the annotation tools together with other omics datasets, such as transcriptomes, proteomes and metabolomes, as already being done in an independent fashion (Marcellin *et al*., 2013). Just as simple as web-based Blast searches, this should happen straightforwardly, without requirements of metabolic modelling expertise. Once a metabolic model becomes available, moreover, as metabolism is diverse and dynamic, more than simplistic two-dimensional representations portrayed by metabolic charts, multiple solutions should be accessible and feasible.

This would allow the functional microbiologist to make biologically detailed and informed decisions when specific aims are pursued. Available phenotypic knowledge, obtained after high-throughput growth conditions and gene knockout screenings, could be considered at this stage. Moreover, although the possibility of accounting for the entire universe of promiscuous enzyme functions encoded by all proteins seems an impossible task, at least at the present time, it should at least be possible to ‘flag’ a potentially highly promiscuous enzyme. For this purpose, the field of chemoinformatics will need to be further developed and become an integral component of post-genomics platforms, as it has occurred with bioinformatics. The potential of interdisciplinary thinking merging chemical and evolutionary principles, as both have sound theoretical foundations, is an attractive possibility.

As computing power has become to be less of a problem, and all research laboratories nowadays have embraced bioinformatics, all this sounds perfectly feasible in computational terms. However, laboratory-based approaches that will mirror the relative efficiency of high-throughput computational analyses are a major pitfall and thus another field of opportunity (Gerlt *et al*., 2011). Just as we have developed the so-called omics techniques, in particular next-generation genome sequencing, there is a need for developing systematic approaches for generating functional data. This, however, will need to go beyond screenings for general biological functions, such as those relying in localization, expression profiles and genetic interactions, to really achieve functional annotation at the molecular level. With the advancement of microfluidics, this appears as an interesting possibility, especially for tackling complex issues as enzyme promiscuity.

In conclusion, starting from a critical assessment of what is a key aspect of current functional post-genomics, namely the way we do functional annotation of genomes, opportunities related to the development of better post-genomics tools could be envisaged. Particularly challenging would be to predict and annotate enzyme promiscuity and moonlighting proteins, but the rewards for integrating dissimilar types of data to tackle this complex problem may be worthy. If this is to be achieved, then metabolic models will not only be accurate, but also they will certainly become a tool for integrated functional annotation. Indeed, as highlighted here, many functional biologists are already doing the integrated analyses needed to overcome some of these problems, so it may be a matter of time for the tools to become universally available.
